# Investigation of *Prototheca bovis* Infection and Its Correlation with Dairy Herd Improvement Data from a Dairy Farm in Central China

**DOI:** 10.3390/vetsci11010037

**Published:** 2024-01-17

**Authors:** Jie Chen, Xiuxiu Hu, Guohong Li, Pingmin Wan, Zhiyong Shao, Erguang Jin, Xiaohua Liu, Qian Yang, Anying Long, Yunguo Qian

**Affiliations:** 1 Institute of Animal Husbandry and Veterinary Science, Wuhan Academy of Agricultural Sciences, Wuhan 430208, China; chenjie@wuhanagri.com (J.C.); huxiuxiu@wuhanagri.com (X.H.); wanpingmin@wuhanagri.com (P.W.); shaozhiyong@wuhanagri.com (Z.S.); jinerguang@wuhanagri.com (E.J.); liuxiaohua@wuhanagri.com (X.L.); yangqian_97@webmail.hzau.edu.cn (Q.Y.); longanying@webmail.hzau.edu.cn (A.L.); 2 College of Animal Science, Yangtze University, Jingzhou 434025, China; 3 Wuhan Keqian Biology Co., Ltd., Wuhan 430206, China; liguohong@kqbio.com; 4 College of Veterinary Medicine, Huazhong Agricultural University, Wuhan 430070, China

**Keywords:** *Prototheca bovis*, dairy cows, mastitis, dairy herd improvement index, milk production

## Abstract

**Simple Summary:**

A multitude of microorganisms contribute to the occurrence of bovine mastitis. In addition to familiar pathogenic microorganisms such as *Streptococcus*, *Staphylococcus*, and *Escherichia coli*, *Prototheca bovis* has attracted more attention in recent years. It is a unicellular, chlorophyll-free, yeast-like algae that thrives in warm, humid environments that are rich in organic matter, resulting in frequent infection of dairy cows. The purpose of this study was to better understand the prevalence of *Prototheca bovis* infection and its potential effects on dairy cow health and milk production. We investigated the prevalence of *Prototheca bovis* in a medium-sized dairy farm in central China. Sample collection was extended for 5 months, from August to December 2022. A variety of pathogenic microorganisms were identified, including *Prototheca bovis*, and its infection rate in raw milk was 50.8–77.5%. Further analysis demonstrated that the infection of *Prototheca bovis* significantly affected the milk yield, somatic cell count, and protein percentage of raw milk. These results indicated that *Prototheca bovis* was one of the main factors causing dairy cow mastitis and posed a high threat to the quality of raw milk. This study offers valuable guidance for efficacious prevention of *Prototheca bovis* infection to dairy farms, such as maintaining a balance between environmental cleaning and humidity, especially during winter seasons.

**Abstract:**

*Prototheca bovis* (*P. bovis*), an alga that has attracted considerable attention over the years as a causative microorganism of mastitis in dairy cows, exhibits limited susceptibility to specific aminoglycosides and antifungal agents, and no effective clinical treatment is currently available, thereby posing challenges for both prevention and treatment. To investigate the infection of *P. bovis* mastitis and its impact on raw milk production, a total of 348 raw milk samples were collected from August to December 2022 from a dairy farm in central China. *P. bovis* and other bacteria were detected, and the average infection rate of *P. bovis* in raw milk was 60.34% (210/348). The total number of colonies and the somatic cell count (SCC) of *P. bovis* positive samples were significantly higher than those of *P. bovis* negative samples (*p* < 0.01). The daily milk yield, 305-day milk yield, peak milk yield, and days to peak milk yield of the *P. bovis* positive samples were significantly lower than those of *P. bovis* negative samples (*p* < 0.01). A correlation analysis showed that *P. bovis* infection was negatively correlated with daily milk yield, 305-day milk yield, peak milk yield, and days to peak milk yield (*p* < 0.0001), while being positively correlated with the total number of colonies, SCC, milk loss, and protein percentage (*p* < 0.0001). These findings may help practitioners in comprehending the occurrence of *Prototheca* mastitis and developing more effective strategies for the prevention of *P. bovis* infections.

## 1. Introduction

*Prototheca* is an achlorophyllous alga belonging to the Chlorellaceae family, although it is sometimes incorrectly referred to as a yeast or fungi [1]. There are more than 15 identified species within the genus *Prototheca*, including *Prototheca ciferri* (formerly *P. zopfii* genotype 1), *Prototheca bovis* (*P. bovis*, formerly *P. zopfii* genotype 2), *Prototheca wickerhamii*, *Prototheca cutis*, and *Prototheca blaschkeae*. These species have been found to cause infections in animals and humans [2,3,4]. *P. bovis* and *P. wickerhamii* maintain the largest host range, including dairy cows, buffaloes, cats, dogs, and goats, and can cause nodular and ulcerative dermatitis in dogs and nasal infection in cats [5,6]. In dairy cows, *P. bovis* mainly causes acute or chronic mastitis, resulting in a swollen udder, decreased milk production, and watery milk, and may be accompanied by flocculent precipitation. The algae thrive in warm, humid environments where there is plentiful organic matter, such as forage, bedding materials, and cattle feces, which has resulted in the frequent infection of dairy cows [7]. In addition, recent studies showed that *P. bovis* isolates are susceptible to several antibiotics and antifungal drugs in vitro, but no effective clinical treatment is currently available, making prevention and treatment more difficult [8,9].

*P. bovis*-caused bovine mastitis was first reported in 1952 [10]. In recent years, cow mastitis caused by *P. bovis* has been reported globally, including in Germany [11], Italy [12,13], Poland [14], Japan [15], Republic of Korea [16], China [17,18,19], and other countries or regions [3,20]. In north China, Shahid et al. identified 84 *P. bovis* isolates from 620 milk samples taken from six large-scale dairy farms in Beijing, Tianjin, and Shandong Province. *P. bovis* was detected in all six dairy farms, with isolation rates ranging from 10% to 18.1% [21]. A recent study showed that the isolation rate of *P. bovis* was as high as 65.0% in raw milk samples, with a somatic cell count (SCC) of more than 1 × 10^6^ cell/mL, while *Streptococcus uberis* and *Staphylococcus aureus* were isolated at 12.5% and 10%, respectively [8]. The possible roles of *P. bovis* infection in dairy cow health and raw milk production are less understood.

In this study, we conducted a 5-month study of *P. bovis* infection and its impact on raw milk production in a dairy farm in central China, aiming to improve our understanding of the effects of *P. bovis* infection on the health of dairy cows and milk production. This heightened knowledge can guide the development of more effective prevention and treatment strategies for *P. bovis* infection.

## 2. Materials and Methods

### 2.1. Study Approval and Sample Collection

The animals were treated and managed according to the Guide for the Care and Use of Laboratory Animals: Eighth Edition (2011) [22] and was approved by the Ethics Committee of Institute of Animal Husbandry and Veterinary Science, Wuhan Academy of Agricultural Sciences (approval No: WHNKYAMO-2021052101; approval date: 21 May 2021).

A total of 348 composite samples of raw milk were collected from a dairy farm located in central China. It is a medium-size farm that occupies a total of 13,000 m^2^ and has a herd of 200 dairy cows, including 120 adult cows. For this study, all 65–75 lactating cows on the farm were sampled on the first day of each month, from August 2022 to December 2022, resulting in approximately 70 samples per month. The collection of raw milk samples comprised equal volumes of milk from each of the four quarters of a cow, completed at the same time that normal milking is performed on the dairy farm. The udders of each cow, particularly the teat ends, were thoroughly cleansed with warm water followed by disinfection using a 0.75% iodine and glycerin solution (DeLaval Tianjin Co., Ltd., Tianjin, China). Gentle massage was applied prior to sampling. Additionally, the initial two to three streams of milk per quarter during each milking session were discarded. Samples were collected in 50 mL sterilized tubes and transported on ice to the laboratory. For each sample, 2 mL was taken for bacterial isolation and colony counting. The antimicrobial bronopol was added to the remaining samples (0.75 mg/mL, final concentration) and used for dairy herd improvement (DHI) analysis. DHI analysis was conducted at the Hubei Livestock and Poultry Breeding Center using MilkoScan 7RM and Fossomatic 7. The daily milk yield per cow was provided by the dairy farm, while the SCC, fat, and protein percentages were determined using MilkoScan 7RM and Fossomatic 7 analyzers. Subsequently, the measured values from the MilkoScan 7RM and Fossomatic 7 data as well as additional data provided by the farm were used to calculate parameters such as peak milk yield, days to peak milk yield, 305-day milk yield, milk loss, and economic loss by using the system for measuring and analyzing dairy cow performance (CNDHI V3.0) [23].

### 2.2. Materials

Two main culture media, tryptic soy agar (TSA) and Sabouraud dextrose agar (SDA), were used for bacterial culture and colony counting. *Prototheca* isolation medium (PIM) was used for *P. bovis* isolation. These media and lactophenol cotton blue staining solutions were purchased from Qingdao Haibo Biotechnology Co., Ltd. (Qingdao, China), and Gram staining solutions were purchased from Zhuhai Beisuo Biotechnology Co., Ltd. (Zhuhai, China).

### 2.3. Isolation and Colony Counting of P. bovis

For accurate counting of *P. bovis* in each sample, the diluted samples (10×, 100×, and 1000×) were streaked on both TSA and SDA plates. Three plates were used for each diluted sample (in triplicate), and 100 μL of sample was used for each plate. The TSA plates were utilized to quantify the total number of bacterial colonies, whereas the SDA plates were employed to enumerate the total number of *Prototheca* colonies. After incubation at 37 °C for 48 h, the colony-forming units (CFU) were counted. Colonies grown on TSA plates had different morphology, size, and color. The most abundant, or dominant, colonies, which consisted of more than 50% of the total colonies, were selected and further purified by streaking on TSA plates. The identification of bacterial species was initially based on colony features, including size, morphology, color, luster, and Gram stain. The suspected *Prototheca* colonies were confirmed with lactophenol cotton blue staining.

### 2.4. Identification of Microorganisms

The detection of bacteria was conducted by 16s rDNA polymerase chain reaction (PCR) and sequencing. The primers used are shown in Table 1. The PCR reaction was as follows: Premix Taq (Ex Version 2.0 plus dye) 12.5 μL, 27-F 1 μL, 1492-R 1 μL, template 1 μL, and RNase-free water supplementation to 25 μL. The PCR program comprised the initial denaturing step at 95 °C for 10 min, then 40 cycles of 30 s at 95 °C, 30 s at 54 °C, and 90 s at 72 °C, followed by the final extension step at 72 °C for 10 min. The PCR amplification products were sent to the Wuhan Tianyi company for sequencing, and the isolates were identified by BLAST comparison in GenBank (https://blast.ncbi.nlm.nih.gov/Blast.cgi, accessed from 10 August to 30 December 2022).

The detection of *P. bovis* was further confirmed by real-time quantitative PCR (qPCR) of the mitochondrial cytochrome b (cob, also named cytB) gene [24]. The primers used are shown in Table 1. The qPCR system was as follows: SYBR Green Premix Ex TaqII 12.5 μL, cob-5-F 0.8 μL, cob-5-R 0.8 μL, template 1 μL, and RNase-free water supplementation to 25 μL. The qPCR program comprised the initial denaturing step at 95 °C for 30 s, then 40 cycles of 5 s at 95 °C, 60 s at 59 °C, and 30 s at 72 °C, followed by the final extension step at 72 °C for 10 min.

If the Ct value was less than 35, the sample was considered to be *P. bovis* positive. If the Ct value was equal to or greater than 40 or there was no amplification curve, the sample was considered to be negative. If the Ct value was greater than 35 and less than 40, the sample was judged as suspicious and was required to be tested twice.

### 2.5. Statistical Analyses

Experimental data were expressed as the mean ± standard error (SE). A *t*-test analysis was performed using SPSS 18.0 (SPSS, Inc., Chicago, IL, USA) and GraphPad Prism 8.0 (GraphPad Software Inc., La Jolla, CA, USA). Sangerbox 3.0 matrix correlation analysis and Spearman’s correlation method in visualization were used for this analysis. *p* < 0.05 was considered statistically different.

## 3. Results

### 3.1. Isolation and Identification of Bacteria and P. bovis

**Figure 1:**
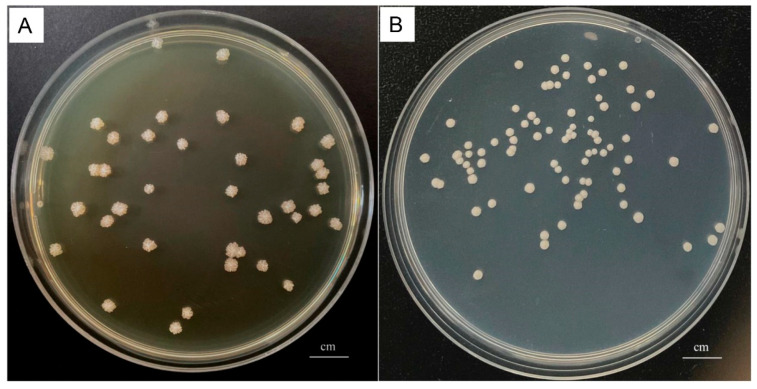
Colony morphology of *P. bovis* on SDA (**A**) and PIM (**B**).

**Figure 2:**
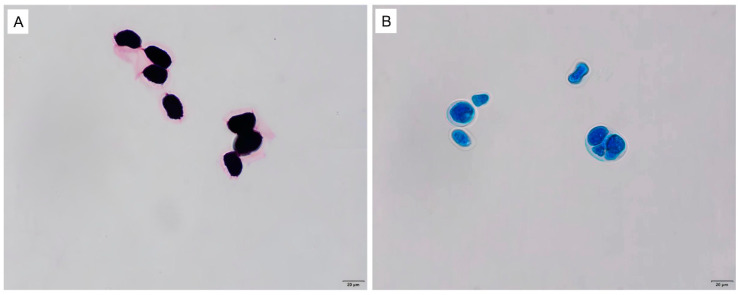
Microscopic morphology of the isolated *P. bovis*. (**A**) *P. bovis* in Gram staining and (**B**) *P. bovis* in lactophenol cotton blue staining.

**Figure 3:**
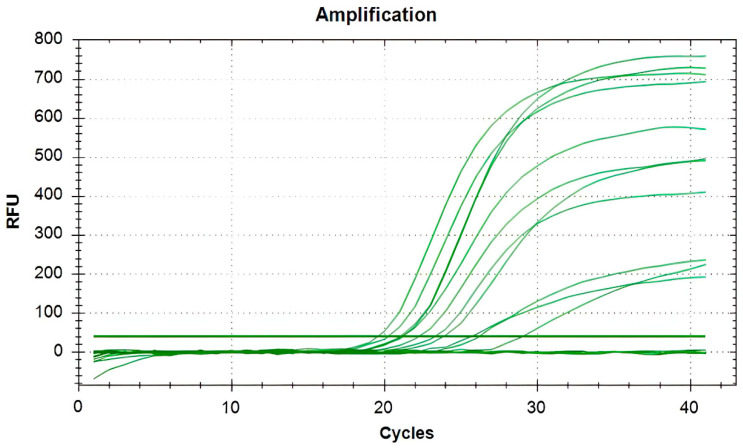
Identification of *P. bovis* by qPCR. Each green line indicates an individual sample. The s-shaped curve indicates samples with a positive amplification for *P. bovis*, and lines near 0 RFU indicate no amplification (negative samples). RFU = relative fluorescence unit.

All milk samples of the dairy farm were aseptically collected and immediately transported to the laboratory for bacteriological analysis. The results showed that the major microorganisms isolated from raw milk were *P. bovis* (210/348), *Streptococcus* spp. (89/348), *Staphylococcus* spp. (53/348), *Acinetobacter* spp. (41/348), and *Corynebacterium* spp.(32/348). Only one bacterium was isolated from approximately 80% of the milk samples, while two to three bacteria were isolated from the remaining samples. Milk samples with two to three bacteria were further tested and confirmed that they were not caused by contamination. *P. bovis* colonies appeared as round and white on both SDA (Figure 1A) and PIM (Figure 1B). Gram staining showed that the *P. bovis* cells appeared spherical or oval in dark purple under the microscope (Figure 2A). Staining with lactophenol cotton blue revealed that a transparent cell wall encircled the outermost layer of the algae (Figure 2B). qPCR of the *cob* gene further confirmed that the isolate was *P. bovis* (Figure 3).

After confirmation of *P. bovis* infection, we analyzed the *P. bovis* infection rates among the collected samples (Table 2). We found that among the 348 raw milk samples, 210 samples were *P. bovis* positive, with a positivity rate of 60.3% (210/348), and the monthly positivity rates were 53.5%, 58.5%, 60.5%, 50.8%, and 77.5% from August 2022 to December 2022, respectively.

### 3.2. Effects of P. bovis Infection on Milk Production

**Figure 4:**
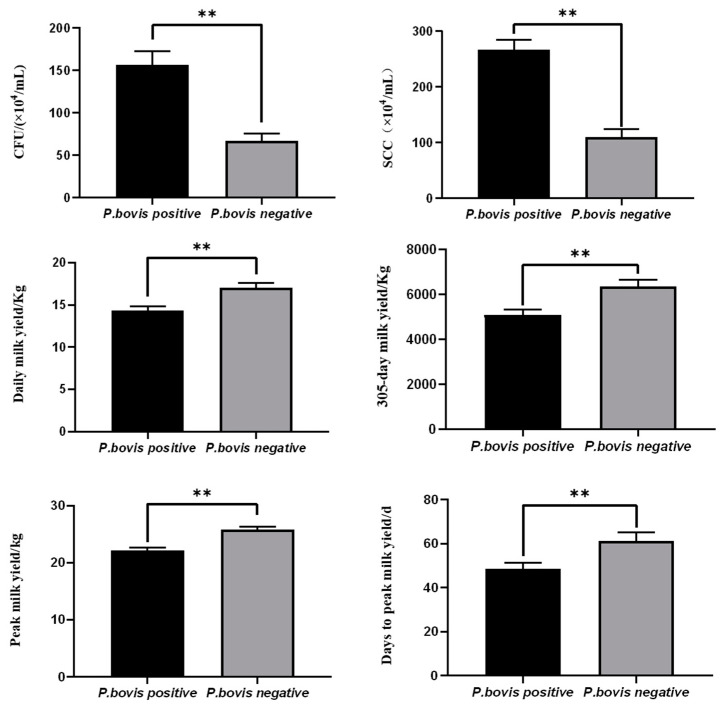
Effects of *P. bovis* infection on main production indicators (** *p* < 0.01).

The possible effect of *P. bovis* infection on milk production was further analyzed. As shown in Figure 4, the average CFU in the TSA plate, representing all bacteria of *P. bovis* positive samples, was 156.38 ± 16.41 × 10^4^/mL, while the average CFU of *P. bovis* negative samples was 67.40 ± 8.33 × 10^4^/mL, demonstrating a statistically significant difference (*p* < 0.01). The average SCC of the *P. bovis* positive samples was 266.80 ± 17.71 × 10^4^/mL, which was significantly higher than that of *P. bovis* negative samples 110.22 ± 14.72 × 10^4^/mL (*p* < 0.01). In addition, the average daily milk yield and 305-day milk yield of the *P. bovis* positive samples (14.37 ± 0.46 kg and 5092.23 ± 211.35 kg, respectively) were significantly lower (*p* < 0.01) than those of *P. bovis* negative samples (17.03 ± 0.55 kg and 6347.17 ± 304.72 kg, respectively). The average peak milk yield and days to peak milk yield of *P. bovis* positive cows were significantly lower than those of *P. bovis* negative cows (*p* < 0.01) (Figure 4). These results indicated that *P. bovis* infection had significant effects on milk production and dairy cow health, including the total number of colonies, SCC, daily milk yield, 305-day milk yield, peak milk yield, and days to peak milk yield.

### 3.3. Correlation Analysis between P. bovis Infection and DHI Data

**Figure 5:**
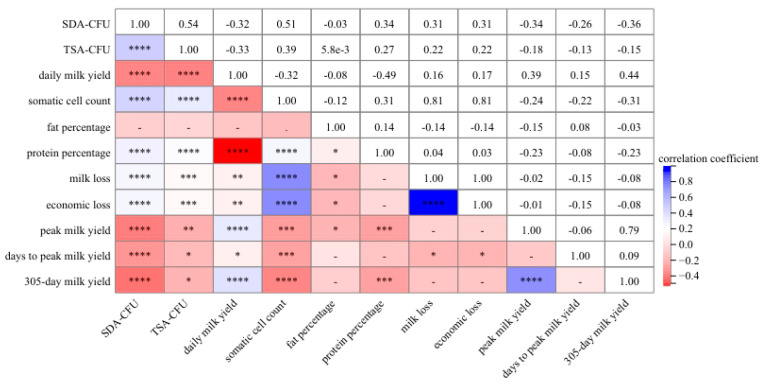
Pearson’s correlation analysis among the colony count of *P. bovis* and DHI data (* *p* < 0.05, ** *p* < 0.01, *** *p* < 0.001, **** *p* < 0.0001). The numbers in the figure are actual *p* values that are not statistically different.

The correlation analysis revealed that the colony count of *P. bovis* (CFUs on SDA medium) in the 348 raw milk samples were negatively correlated with the daily milk yield (*r* = −0.32, *p* < 0.0001), 305-day milk yield (*r* = −0.36, *p* < 0.0001), peak milk yield (*r* = −0.34, *p* < 0.0001), and days to peak milk yield (*r* = −0.26, *p* < 0.0001) (Figure 5). Meanwhile, there was a significantly positive correlation between the colony count of *P. bovis* and the total number of colonies (all bacteria, CFUs on TSA medium) (*r* = 0.54, *p* < 0.0001), SCC (*r* = 0.51, *p* < 0.0001), milk loss (*r* = 0.31, *p* < 0.0001), and protein percentage (*r* = 0.34, *p* < 0.0001). There was a negative correlation between the colony count of *P. bovis* and fat percentage (*r* = −0.03), but this difference was not statistically significant (*p* > 0.05).

## 4. Discussion

It was reported as early as 1952 [10] that *P. bovis* causes mastitis in dairy cows, which is a complex mammary gland disease that affects the health of dairy cows and milk production [25]. In Italy, a large-scale screening survey involving 24 dairy farms showed that *P. bovis* played an important role in dairy cow mastitis. A total of 161 *Prototheca* spp. isolates isolated from 3208 milk samples and 411 barn-surrounding environmental samples of those dairy herds were characterized. Of the 108 *Prototheca* isolates from composite milk samples, 97.2% were characterized as *P. bovis* [26]. A study conducted in southeast Poland showed that *P. bovis* was the third most common pathogen, with an overall incidence of only 4.6%. It is worth noting that the average SCC in the *Prototheca*-containing milk was 4.02 × 10^6^ cells/mL, whereas it was 0.13 × 10^6^ cells/mL in the *Prototheca*-free milk [14]. In China, *P. bovis* was first reported in dairy cows in 2011, with the isolation rate as high as 72.7% from clinical mastitis [27]. More studies revealed that the spread of *P. bovis* infection in China is extensive, from large- to small-scale dairy farms, and from dairy cows to their surrounding environment [8,18,21].

In this study, we investigated the *P. bovis* prevalence in a medium-sized dairy farm in central China. Sample collection was extended for 5 months, from August to December 2022. August and September, when the weather is relatively hot and humid, are usually considered to have a higher incidence of mastitis in dairy cows. Our results showed that from August to October, the detection rate of *P. bovis* increased gradually from 53.3% to 60.5%, and then dropped to 50.8% in November, which suggested a possible relationship between the *P. bovis* infection rate and weather conditions. The average high temperature in Hubei province, where the dairy farm is located, was 34 °C in August 2022, with a maximum temperature of 39.7° C. The average high temperature and maximum temperature in September were 31 °C and 37 °C, respectively. The highest temperature in October, 40.6 °C, occurred on 3 October 2022, and our October sample was collected on 1 October. The average high temperature in November dropped to 16 °C. However, the highest monthly infection rate reached 77.5% in December 2022, despite an average high temperature of only 10 °C. In Hubei Province, where the dairy farm is located, the weather is cold and rainy from the end of November to the following January. Consequently, dairy farms often curtail the frequency of environmental cleaning and disinfection during inclement winter weather to mitigate potential increases in humidity levels, which could foster the proliferation of *P. bovis* and other microorganisms within dairy farm environments, thereby heightening the susceptibility of dairy cows to contracting mastitis from their surroundings. It is, therefore, important to maintain a good balance between environmental cleaning and humidity levels, especially during cold seasons.

Microbial infection is a major cause of dairy cow mastitis, the most influential factor affecting milk production [28]. Besides *P. bovis*, the main pathogenic microbes include *Streptococcus*, *Staphylococcus*, *Escherichia coli*, *Corynebacterium*, and *Acinetobacter* [29,30]. In this study, we employed 16S rDNA sequencing and *P. bovis* qPCR to identify several microorganisms in the raw milk samples that were collected, including *P. bovis*, *Streptococcus* spp., *Staphylococcus* spp., *Acinetobacter* spp., and *Corynebacterium* spp., among which *P. bovis* was one of main pathogens isolated. This result was similar to several previous studies in Asia and Europe [8,14,26,27]. In all cases, *P. bovis* was identified as a main pathogen that severely threatened the health of dairy cows. Further extended epidemiological investigations would help us better understand the occurrence of as well as prevention strategies for *P. bovis* infection.

The clinical symptoms of *P. bovis* mastitis are lacking in specificity, and laboratory diagnosis is required when algal infection is suspected. Traditionally, *P. bovis* identification involves multiple steps, the first of which is to culture and isolate the suspected *P. bovis* using TSA, SDA, and PIM media. The isolates are then determined through Gram staining and lactophenol cotton blue staining, then examined with a microscope, followed by further identification using molecular biological methods. Jagielski et al. [2] developed a PCR method based on the rDNA sequence of *Prototheca* spp. that can distinguish three different genotypes of *Prototheca*. Marques et al. [31] established a method based on the sequence of the internal transcribed spacer (ITS) in ribosomal DNA. It was successfully applied for the identification of *P. bovis* and *P. blaschkeae* in bovine milk. The recently developed multiplex TaqMan qPCR system can diagnose several species of *Prototheca* rapidly and accurately. The method was based on the specific sequences of AccD (encoding acetyl CoA reductase) for *P. bovis*, cox1 (encoding cytochrome C oxidase subunit 1) for *P. wickerhamii*, cytB (encoding cytochrome B) for *P. blashkeae*, and atp6 (encoding transporting ATPase F0 subunit 6) for *P. ciferrii* [32]. In this study, we used a qPCR method based on the primers of cob-5, which was designed for the cytb gene of *P. bovis*, with excellent specificity and sensitivity. The detection limit of this method is 20.9 CFU/mL [24]. In addition to using purified bacterial liquid as a template, this method can also detect milk samples that have been simply treated with high-speed centrifugation and PBS washing [24], which can greatly shorten the clinical diagnosis time and has obvious advantages in the early and rapid diagnosis of *Prototheca* mastitis.

DHI testing is a standard measurement in the dairy industry for evaluating the milk production of dairy cows [33]. It evaluates multiple factors, such as the milk SCC, total milk yield, and quality of milk (protein, fat, dry matter, etc.) to determine the quality of milk and the health of dairy cows. In this study, the dairy farm we sampled had participated in DHI testing for many years, which allowed us to compare the detection of *P. bovis* with DHI data for statistical analysis. We found that there were significant differences in the total number of colonies, SCC, milk yield, and other DHI indexes between *P. bovis* positive and *P. bovis* negative cows. This is consistent with a previous study, which showed that *P. bovis* infection significantly decreased the milk quality, including milk yield, fat, and lactose [34]. It is worth noting that the average SCC of *P. bovis* positive milk samples was 266.80 ± 17.71 × 10^4^/mL, while the average SCC of milk samples without *P. bovis* infection was 110.22 ± 14.72 × 10^4^/mL. This exceeded the SCC standard of the Chinese dairy enterprises (4 × 10^5^/mL), recommended by the China Dairy Association (T/DACS 001-2021). Further investigation is needed to determine whether this was related to microbial infections such as *Streptococcus* and *Staphylococcus* or to other management factors. Upon our analysis, the owners of the farm had decided to remove the dairy cows with high SCC and CFU levels from commercial milk production, and these cows were treated for mastitis. Our correlation analysis further demonstrated that *P. bovis* infection was negatively correlated with daily milk yield, 305-day milk yield, peak milk yield, and days to peak milk yield, and was positively correlated with the total number of colonies, SCC, and protein percentage. The results clearly indicated that *P. bovis* infection played an important role in dairy cow health and raw milk quality.

Studies have shown that *P. bovis* can invade the mammary gland parenchyma [35]. After being infected with *P. bovis*, increased expression of interleukin-1β, C-X-C motif chemokine ligand 1 (Cxcl-1), and tumor necrosis factor-α (TNF-α) were observed in mouse macrophages and mammary epithelial cells [36]. The inflammatory response could be further activated through mitochondrial ROS [37]. Therefore, the treatment of *P. bovis* infection can be designed to directly kill the pathogen and control the inflammatory response. So far, only a few chemical drugs have been developed to be effective in inhibiting the growth of *P. bovis*, leaving ranchers few options for treatment of *P. bovis* infection [8]. To explore more treatment options, our lab is conducting a study on the efficacy of traditional Chinese medicine against *P. bovis*. Our findings suggest that certain active components in Chinese medicine exhibit significant inhibitory effects on the growth of *P. bovis* (Chen, unpublished data).

Environmental control is also an important element in the control of *P. bovis* infection. In a recent study, we detected a certain amount of *P. bovis* in the dairy farm environment, including the manger, dairy bed (where the cow rests), and fermentation padding, which is composed with agricultural straw, saw powder, rice husk, and fermentation bacteria. The fermentation padding is used for dairy manure fermentation and degradation. The farm environment also includes drinking pool edge, feed truck, manure shovel truck, and milking parlor ground, where the cows are held for milking [38]. It was suggested that effective cleaning and disinfection can control the propagation of *P. bovis* in the environment. Iodine solution, sodium hypochlorite, chlorhexidine, and peracetic acid could effectively inhibit the growth of *P. bovis* [39,40,41].

## 5. Conclusions

In this study, a medium-scale dairy farm in central China was monitored for 5 months. *P. bovis* infection was investigated monthly and was used for statistical analysis along with DHI data. It was found that *P. bovis* was the main pathogenic microorganism that significantly affected the SCC, milk yield, milk loss, and other DHI indexes of dairy cows. Our study provides important information for better monitoring and prevention strategies for *P. bovis* infections. One limitation of this study is its relatively small sample size. The study was conducted using samples obtained from only one medium-sized farm. Large-scale epidemiological investigations are needed to better understand the level of infection that leads to clinical outbreaks of *P. bovis*. These investigations will help develop comprehensive measures, along with the development of sensitive drugs and further refinement of management measures such as environmental disinfection, which will mitigate the adverse effects of *P. bovis* infection on dairy cow health and milk quality.

## Figures and Tables

**Table 1:** The sequences of the primers used in this study.

Primer	Sequence (5′-3′)	Target Fragment Size
16s rDNA 27-R	AGAGTTTGATCCTGGCTCAG	1500 bp
16s rDNA 1492-F	TACGGCTACCTTGTTACGACT	
cob-5-F	CTAGTTATTCAAGTCCTCG	131 bp
cob-5-R	AATTACTGTAGCACCCC	

**Table 2:** *P. bovis* infection rate of composite raw milk samples.

Month of Year in 2022	Number of Samples	*P. bovis* Positive Samples	*P. bovis* Negative Samples	*P. bovis* Positivity Rate (%)
August	71	38	33	53.5
September	65	38	27	58.5
October	76	46	30	60.5
November	65	33	32	50.8
December	71	55	16	77.5
Total	348	210	138	60.3

## Data Availability

The datasets generated and/or analyzed during the current study are available from the corresponding author on reasonable request.
